# Chronic exertional compartment syndrome of the forearm diagnosed in a wider spectrum of patients and not seldom with a history of the same diagnosis in the lower legs

**DOI:** 10.1002/jeo2.70017

**Published:** 2024-09-18

**Authors:** Louise Sjöcrona, Sophia H. Lindorsson, Kajsa Rennerfelt

**Affiliations:** ^1^ Department of Orthopaedics, Institute of Clinical Sciences, Sahlgrenska Academy University of Gothenburg Gothenburg Sweden; ^2^ Department of Orthopaedics Sahlgrenska University Hospital Gothenburg Sweden; ^3^ Capio Spine Center Gothenburg Sweden

**Keywords:** chronic compartment syndrome, chronic exertional compartment syndrome, forearm pain, intracompartmental pressure, intramuscular pressure

## Abstract

**Purpose:**

To identify patient characteristics associated with forearm chronic exertional compartment syndrome (CECS) and to demonstrate the distribution of intramuscular pressure (IMP) values at 1 min postexercise in a cohort of patients with exercise‐induced forearm pain.

**Methods:**

A consecutive series of 99 patients seeking orthopaedic consultation for chronic exertional forearm pain underwent IMP measurements between 2010 and 2023. The diagnosis of CECS was confirmed (*n* = 34) or ruled out (*n* = 65) based on the patient's history, clinical examination and IMP measurements.

**Results:**

There were significantly more male patients in the CECS group than in the group of patients where the diagnosis was ruled out. Furthermore, a significantly higher proportion of the CECS patients had been previously treated for CECS of the lower legs. The most common occupation was office worker (21%), followed by craftsperson (18%). The most common main physical activities were strength training (21%) and cycling (15%). The median (range) 1‐min postexercise IMP values for patients with CECS were 34 (23–68) mmHg for the flexor compartment and 32 (25–67) mmHg for the extensor compartment.

**Conclusion:**

This study demonstrates a more general population of CECS patients compared to previous studies. Notably, more than a fifth of the CECS patients had previously been treated for CECS in the lower legs. Importantly, considering the 95% confidence interval for IMP values in patients without CECS, the most used IMP cutoff value for diagnosing CECS appears to be too high for the forearm compartments.

**Level of Evidence:**

Level II.

**Abbreviations**
BMI: body mass index
CECS: chronic exertional compartment syndrome
CI: confidence interval
IMP: intramuscular pressure
non‐CECS: patients without CECS
SD: standard deviation

## INTRODUCTION

Chronic exertional compartment syndrome (CECS) of the forearm presents as muscle pain, tightness in the forearm muscles and hindrance of muscle function during activity [7]. The symptoms are absent at rest. The condition is thought to be rare in the general population; patients with CECS of the forearm have earlier been described as exposed to manual work involving the hands or to heavy armload during sports [7]. The symptoms of CECS occur bilaterally in 70%–100% of the patients [4, 29].

The aetiology of CECS is not clear, but overuse activity is considered a contributory factor [21]. Anatomical factors such as limited compartment size, constricted fascia or reduced fascial elasticity may contribute to the cause of CECS [12]. Observations have been made that some patients with CECS show signs of inheritance [9].

Since the condition is rare, most of the published articles about CECS of the forearm are based on case reports or small study populations [7, 11, 21, 28]. Studies including larger study populations on CECS of the forearm focus on diagnostic methods, surgical treatment options and postoperative outcomes, many of them based on numerous study populations from previous studies [3, 4, 24, 29]. Overall, CECS of the upper extremity is sparsely described in literature compared to CECS of the lower extremity, and the diagnosis might be overlooked clinically in the general population.

The demography of patients described in previous studies has shown a spectrum of active motorcyclists [4, 10], gymnasts [30], wheelchair athletes [32], climbers [19], rowers [11] and patients with heavy arm workload [23, 25, 32]. Overall, patients with CECS of the forearms are usually exposed to repetitive isometric muscle load of the wrist [11]. Earlier studies have suggested that men are predisposed for forearm CECS [11, 14], and a recent systematic review show that 98.5% of the patients with forearm CECS were male [29]. It is not known if the risk factors among men are anatomical or environmental, nor if the diagnosis is being overlooked in women.

The diagnosis of CECS of the forearm is based on the patient's history, clinical examination and invasive measurements of intramuscular pressure (IMP) following an exercise test. IMP measurement performed by an experienced clinician is generally considered to be safe, but as it is an invasive investigation, complications may occur [13]. The arterial line manometer, connected to a side‐port needle or a slit catheter, is the most accurate device to use in the measurement of IMP [5].

The most widely used IMP criteria for CECS are based on the Pedowitz diagnostic criteria, where the IMP cutoff values for all muscle compartments and both genders are set at ≥15 mmHg pre‐exercise, ≥30 mmHg at 1 min postexercise or ≥20 mmHg at 5 min postexercise [22]. The 1‐min postexercise IMP has been suggested to be the best measure [2]. There have been suggestions that it would be accurate to lower the 1‐min postexercise IMP cutoff value for diagnosing CECS of the forearm [3, 19, 31]. However, there is a lack of studies investigating the distribution of IMP values at 1 min postexercise in patients with forearm CECS.

Anatomically the muscles of the forearm can be divided into four compartments: the extensor muscles compartment, the superficial flexor muscles compartment, the deep flexor muscles compartment and the mobile wad of Henry. However, most studies on CECS in the forearm focus on IMP measurements of two compartments—the flexor compartment, including both the superficial and the deep flexor muscle compartments, and the extensor compartment [1, 6, 8, 11].

The treatment for forearm CECS is fasciotomy of the flexor compartment and/or the extensor compartment. Fasciotomy has demonstrated satisfactory results in the treatment of forearm CECS [4, 7, 10, 15, 28].

The objective of this study is to identify patient characteristics associated with forearm CECS and to demonstrate the distribution of IMP values at 1 min postexercise in a cohort of patients with forearm pain during exercise.

## MATERIALS AND METHODS

The study protocol was approved by the regional ethics committee (ID number 589‐18 and 2023‐00535‐02). The study population was a consecutive series of 99 patients (80 men and 19 women) with a median age of 33.5 years (range 19–56 years), who suffered from exercise‐induced forearm pain and experienced clinical signs of CECS. The patients were referred to the Orthopaedic department at Sahlgrenska University Hospital, Gothenburg, Sweden, between 2010 and 2023 for an evaluation of suspected CECS of the forearm. Prior to the appointment, all patients filled out a form, including their characteristics, history of pain and activity parameters. The appointment included an exercise test aimed to induce their symptoms followed by IMP measurements as part of the consultation.

The diagnosis of CECS was either confirmed or ruled out based on the patient's history, clinical examination and invasive measurement of IMP after an exercise test. The diagnostic criteria for CECS of the forearm included (1) exercise‐induced forearm pain with a reversal of symptoms at rest, (2) swelling and tenderness of the forearm immediately after exercise, (3) impaired muscle function during activity and (4) IMP measurement of ≥30 mmHg at 1 min after exercise or ≥20 mmHg at 5 min after exercise [22]. All four criteria were required for the diagnosis of CECS.

### Clinical appointments

At the clinical appointment, the medical history was collected, and a clinical examination was performed by one of three orthopaedic surgeons with several years of experience in CECS. Information about patient characteristics (sex, age, BMI and occupation), pain (duration and local) and physical activity was obtained from the forms. The occupations were divided into three subcategories: (1) heavy arm workload (craftspeople, vehicle technicians, assemblers, warehouse workers, professional drivers, fishers, construction workers, sailors and massage practitioners); (2) likely arm workload (service and electrical technicians, healthcare workers, police officers, chefs, property managers and park rangers); and (3) no arm workload (students, teachers, office workers, priests, nonworkers and unknown occupation). Additionally, the activity at the time of onset of symptoms was also classified into three subcategories: (1) heavy arm‐loading training (motorsports, boxing, racket sports, rowing, sailing); (2) likely arm‐loading training (general training, strength training, yoga, fishing, floorball and swimming) and (3) no arm‐loading training (no training, horse riding, going for walks and physiotherapy).

### Exercise test

The exercise test was individualized to elicit the patient's forearm pain and muscle dysfunction. In most cases, the exercise included repetitive hand squeezing of an elastic rubber ball, repeated flexion and extension of the wrist holding a 0.5–1 kg dumbbell and repeated gripping of a hand‐grip exercise tool. The patients performed the exercise test until they were unable to continue due to muscle exhaustion and/or forearm pain.

### Monitoring IMP

A microcapillary infusion system (Hemo 4; Siemens) and monitor (SC9000; Siemens) was used to measure IMP in the affected compartment/s in the most symptomatic forearm 1 min after the exercise test [17, 18, 26]. The extensor compartment and/or the flexor compartment was measured. With the patient in a relaxed position, sitting or supine, the forearm was placed on a table with the elbow slightly flexed and the wrist in a neutral position. Elastic soft supports (ESWELL, Simonsen & Well, Denmark) were placed under the elbow and the wrist to avoid external compression. The skin was first penetrated by a needle (diameter 1.2 mm) to avoid occlusion of the IMP needle. An IMP needle with four side holes was then inserted at the same site at a 30‐degree angle to the long axis of the forearm in a distal direction into the muscular compartment, parallel with the fibres of the muscles. The IMP needle was connected to a transducer line (length 150 cm) filled with saline, which was linked to the pressure system before insertion. An infusion of 0.9% saline solution was used through the system and out at the tip of the needle, with an infusion rate of 0.2 mL/h, to maintain the bulging of fluid at the tip of the needle. The pressure recording system was calibrated before each measurement.

IMP values were measured in the clinically symptomatic compartment/s of the forearm, where the pain was localized and/or the muscles were tight on palpation. In patients in whom both the flexor and the extensor compartments were measured, the most symptomatic compartment was measured first. In patients with bilateral symptoms, only the most symptomatic arm was measured. The 1‐min postexercise IMP values are presented in the present study. IMP values 5‐min postexercise were recorded in patients whose IMP values 1‐min postexercise were between 20 and 29 mmHg; however, these were obtained from only a small number of patients (four patients).

### Statistical analysis

The IMP data from the two compartments of the forearm were not normally distributed in patients with CECS, but they were normally distributed in patients without CECS. Results are presented as median and range. Mean IMP values with standard deviation (SD) in patients without CECS were calculated to determine the 95% confidence interval (CI). Frequency counts and percentages have been used for categorical variables. Continuous variables between groups were compared using the Mann–Whitney *U*‐test, and Pearson's Chi‐Square test was used for categorical variables. Significance was set at *p* < 0.05. Analyses were performed using IBM SPSS software version 26 (IBM Corp.).

## RESULTS

### Differences in characteristics of patients with CECS and those without CECS

The majority of the 99 patients admitted for suspicious CECS were men in their thirties, who had had their bilateral symptoms for ≥24 months, from both the flexor and the extensor compartments. Of these 99 patients, 34 were diagnosed with CECS according to the Pedowitz diagnostic criteria. There were significantly (*p* = 0.015) more male patients in the CECS group than in the group of patients where the diagnosis was ruled out. Furthermore, a significantly (*p* = 0.004) higher proportion of the CECS patients had been previously treated for CECS of the lower legs (Table 1).

**Table 1:** Characteristics of the patients with chronic exertional compartment syndrome (CECS) and those without CECS (non‐CECS).

Variable	CECS	Non‐CECS	*p*‐Value
*N*	34	65	
Sex, *n* (%)			**0.015** c
Male	32 (94)	48 (74)	
Female	2 (6)	17 (26)	
Age in years, median (range)	33 (19–53)	34 (19–56)	0.439^d^
BMI in kg/m^2^, median (range)^a^	26 (20–34)	25 (20–38)	0.235^d^
Symptom duration ≥24 months, *n* (%)	23 (68)	40 (62)	0.549^c^
Bilateral symptoms, *n* (%)	29 (85)	47 (72)	0.146^c^
Affected compartments, *n* (%) b			0.192^c^
Flexor	11 (32)	15 (23)	
Extensor	2 (6)	12 (19)	
Flexor and extensor	21 (62)	37 (58)	
CECS also in the legs, *n* (%)	7 (21)	2 (3)	**0.004** c
Occupation, *n* (%)			0.328^c^
Heavy arm workload	16 (47)	21 (32)	
Likely arm workload	4 (12)	12 (19)	
No arm workload	14 (41)	32 (49)	
Physical activities, *n* (%)			0.651^c^
Heavy arm‐loading training	9 (26)	12 (18)	
Likely arm‐loading training	18 (53)	38 (59)	
No arm‐loading training	7 (21)	15 (23)	

*Note*: Alpha level was set at *p* < 0.05, which is indicated in bold.

Abbreviation: BMI, body mass index.

^a^ CECS: *n* = 34 (32 men, 2 women), non‐CECS: *n* = 60 (45 men, 15 women).

^b^ CECS: *n* = 34 (32 men, 2 women), non‐CECS: *n* = 64 (47 men, 17 women).

^c^ Chi‐square test.

^d^ Mann–Whitney *U* test.

### Occupation and physical activity in CECS patients

The occupation and the main physical activity at the onset of symptoms in patients who were diagnosed with CECS, based on the currently used diagnostic criteria, are presented in Table 2. The most common occupation was office worker (*n* = 7 [21%]), followed by craftsperson (*n* = 6 [18%]) and student (*n* = 4 [12%]). The most common main physical activities were strength training (*n* = 7 [21%]), cycling (*n* = 5 [15%]), motor sport (*n* = 4 [12%]) and general training (*n* = 4 [12%]). Among the CECS patients, five patients (15%) had a heavy arm workload and practised heavy arm‐loading training.

**Table 2:** Occupation and main physical activity of the patients with chronic exertional compartment syndrome (CECS) of the forearm.

	CECS (*n* = 34)
Occupation, *n* (%)	
Office worker	7 (20.6)
Craftsperson	6 (17.6)
Student	4 (11.8)
Assembler	3 (8.8)
Professional driver	2 (5.9)
Vehicle technician	2 (5.9)
Construction worker	1 (2.9)
Warehouse worker	1 (2.9)
Other occupation	8 (23.5)
Main physical activity, *n* (%)	
Strength training	7 (20.6)
Cycling	5 (14.7)
Motor sport	4 (11.8)
General training	4 (11.8)
Climbing	3 (8.8)
None	3 (8.8)
Boxing	3 (8.8)
Racket sport	2 (5.9)
Other type of training	2 (5.9)
Unknown	1 (2.9)

### One‐minute postexercise IMP values in the two forearm compartments

IMP measurements were performed in all 99 patients. The 1‐min postexercise IMP values of the affected compartments in patients with CECS and of all compartments measured in patients without CECS are presented in Table 3. The median IMP values for patients with CECS were 34 mmHg for the flexor compartment and 32 mmHg for the extensor compartment (Table 3). There were four patients diagnosed with CECS who had a 1‐min postexercise IMP <30 mmHg. The CECS diagnosis in these patients was based on IMP values ≥20 mmHg 5‐min postexercise, in addition to the patient's history and the clinical findings.

**Table 3:** Intramuscular pressure (IMP) at 1‐min postexercise in patients with chronic exertional compartment syndrome (CECS) and without CECS (non‐CECS).

	Median		Interquartile range		Min–Max	
	IMP (mmHg)		IMP (mmHg)		IMP (mmHg)	
Compartment	CECS	Non‐CECS	CECS	Non‐CECS	CECS	Non‐CECS
Flexor^a^	34	13	31–36	9–17	23–68	2–23
Extensor^b^	32	10	30–44	7–14	25–67	3–23

^a^ CECS: *n* = 31, non‐CECS *n* = 50.

^b^ CECS: *n* = 15, non‐CECS *n* = 51.

**Figure 1:**
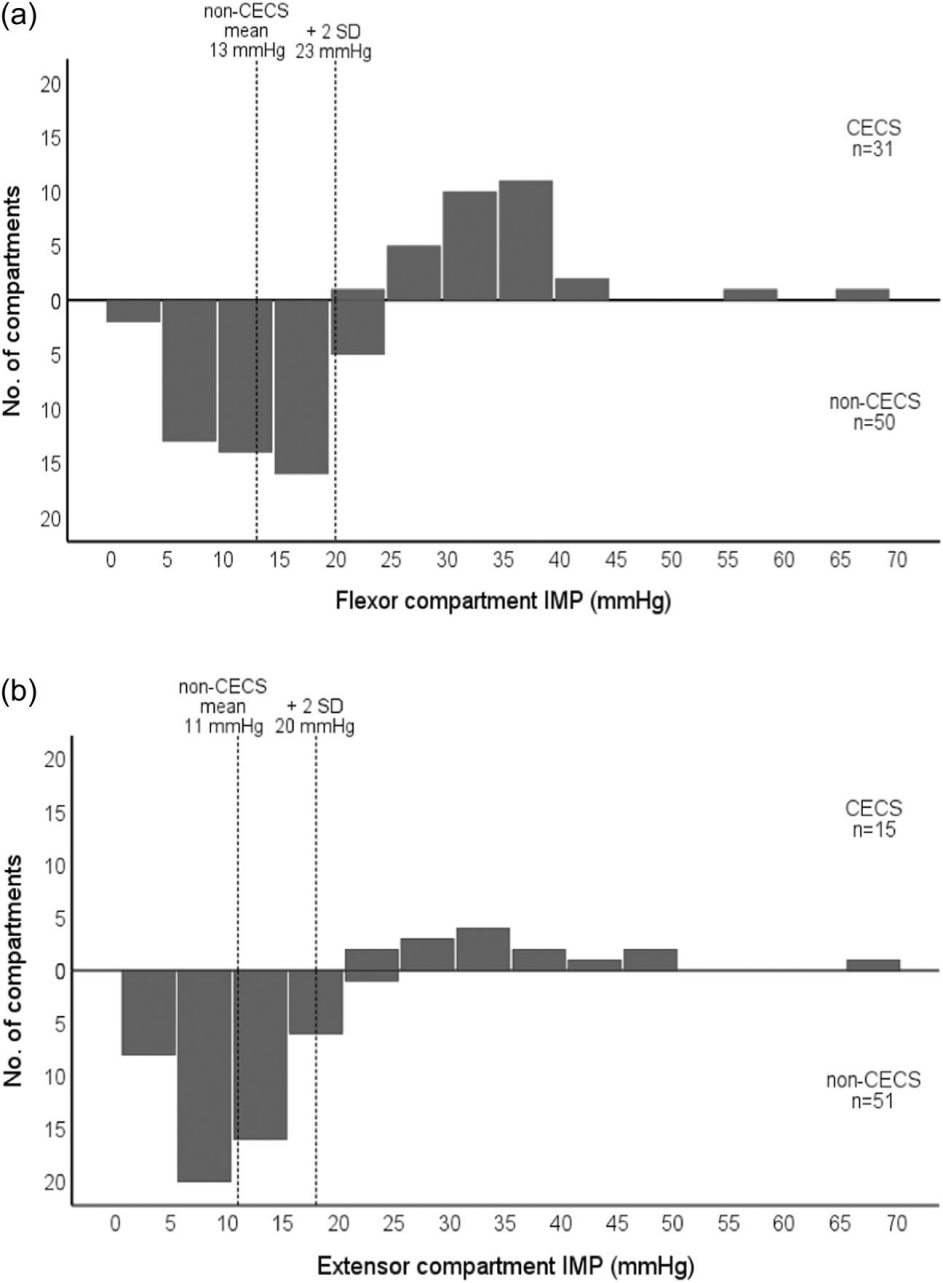
Distribution of 1‐min postexercise intramuscular pressure (IMP) values in the two forearm compartments. Distribution of IMP values 1‐min postexercise in affected flexor compartments (a) and extensor compartments (b) of 34 patients with chronic exertional compartment syndrome (CECS) and in all compartments measured in 65 patients without CECS (non‐CECS). In each histogram, the mean IMP value and the mean plus 2 standard deviations (2 SD) are shown for the patients without CECS.

The distributions of the 1‐min postexercise IMP values of the affected compartments in patients with CECS and of all compartments measured in patients without CECS are presented in Figure 1. The mean IMP value and the mean plus two SD are shown for the compartments in the patients for whom the CECS diagnosis was ruled out.

## DISCUSSION

The main finding of this large cohort study of patients with suspected CECS of the forearm was that among the patients with CECS, 21% were previously treated for CECS of the lower leg. Moreover, the group of patients diagnosed with CECS (*n* = 34) consisted of 94% men, among whom the most common occupations were office work and craftsperson, and the most common forms of physical activities were strength training and cycling. In addition, the median 1‐min postexercise IMP values in the flexor compartment and the extensor compartment in patients with CECS were 34 and 32 mmHg, respectively. Based on analyses of IMP values in patients without CECS, a lowering of the cutoff value for diagnosing CECS in the forearm can be further investigated.

In the present study, the most common occupations among patients with CECS were office work and craftsperson, almost equally distributed. Moreover, general strength training at the gym and cycling were the most common physical activities. These findings demonstrate a more general population of CECS patients compared to previous studies, in which patients were involved in heavy physical work or in specific sports with heavy arm muscular load, such as motorcycling, climbing and rowing [11, 23, 24]. The findings of the present study can enlarge the spectrum of patients who could potentially suffer from CECS of the forearm and thereby decrease the proportion of patients being overlooked.

The patients diagnosed with CECS in the forearm in this study constitute a group of 32 men and 2 women, and the majority of men is in line with previous studies [11, 14, 29]. In the lower leg, the presence of CECS is more equally distributed regarding gender [16, 26, 27]. It is not clear whether the risk factors of CECS of the forearm are anatomical or environmental. The presented male predisposition for CECS in the upper extremity in the literature might have led to the diagnosis being overlooked in women.

Out of the 99 consecutive patients referred for evaluation of suspected forearm CECS, the diagnosis was confirmed in approximately one‐third of the patients. Both patients with CECS and those without CECS predominantly experienced bilateral symptoms from both flexor and extensor compartments. These findings are in line with the findings of Harrison et al. [11]. CECS of the forearm is a rare diagnosis, and few practitioners may be familiar with the symptoms. There is a wide range of differential diagnoses, such as carpal tunnel syndrome, epicondylitis and nerve entrapments, which include symptoms that might resemble CECS symptoms [8]. The present study shows that the majority of the CECS patients had had their symptoms for ≥24 months, which could be due to the large number of differential diagnoses, and thereby a delay of the medical profession.

A somewhat surprising finding was that 21% of the patients with forearm CECS had been previously diagnosed with CECS in the lower extremity. No other studies are at this point available to show the link between CECS in different anatomical locations. As the aetiology of CECS is not clear, the link between CECS in the forearm and the lower leg can only be speculated on. The anatomical factors suggested to contribute to the cause of CECS, such as limited compartment size, constricted fascia or reduced fascial elasticity, might be general for all muscle compartments in CECS patients. Further research is needed, including analyses of fascia from different anatomical locations in patients with CECS. However, the awareness of this possible connection can facilitate the investigation and hopefully shorten the time for diagnosis.

Considering the IMP, the median 1‐min postexercise IMP values for patients with CECS were 34 mmHg for the flexor compartment and 32 mmHg for the extensor compartment in the present study. Since 1990, the Pedowitz diagnostic criteria for CECS have been widely used, with the cutoff IMP value of ≥30 mmHg at 1 min postexercise or ≥20 mmHg at 5min postexercise [22]. These criteria are suggested for all compartments in all anatomical regions. The Pedowitz diagnostic criteria were based on calculations of the mean IMP value for the anterior compartment of the lower leg plus two SDs in patients who were not diagnosed with CECS. In the present study, the IMP values recorded in 50 flexor and 51 extensor compartments in patients with no CECS showed mean IMP 1‐min postexercise plus two SD (representing the 95% CI) of 23 and 20 mmHg, respectively. Based on these findings, the most used 1‐min postexercise cutoff IMP value of 30 mmHg appears to be too high for the forearm compartments, suggesting the consideration of lower cutoff values. This observation is supported by earlier studies of patients with CECS in the forearm, which propose a lower cutoff value for diagnosing CECS of the forearm [3, 19, 31]. A 1‐min postexercise cutoff IMP value of 25 mmHg for the forearm compartments could be proposed. However, further investigations are needed to potentially lower the IMP cutoff value for diagnosing forearm CECS**.** Future studies within this area are especially important for female patients with exercise‐induced forearm pain, since it has been shown that men have higher IMP values than women, both among CECS patients and among healthy individuals [16, 20, 26].

One strength of the present study was the inclusion of such a large cohort of patients with exertional forearm pain. All patients underwent clinical examinations and IMP measurement in a standardized manner, conducted by one of three experienced clinicians at the same clinic. However, a limitation of the present study is the lack of a healthy control group. As measurement of IMP is an invasive method with associated risks, there are ethical difficulties in performing such a study in a large cohort. Additionally, IMP values were measured only in symptomatic compartments, due to the risk of complications associated with invasive measurements [13].

## CONCLUSION

This study demonstrates that patients diagnosed with CECS are predominantly men, working in an office or as craftspeople, and actively practising strength training or cycling. Notably, more than a fifth of the CECS patients had been previously treated for CECS in the lower legs. Importantly, the median 1‐min postexercise IMP values in patients diagnosed with CECS were below 35 mmHg in both the flexor and the extensor compartment of the forearm, and the cutoff value for diagnosing CECS appears to be too high for the forearm compartments.

## AUTHOR CONTRIBUTIONS

Louise Sjöcrona, Sophia H. Lindorsson and Kajsa Rennerfelt contributed to the design and the implementation of the research, to the analysis of the results and to the writing of the manuscript.

## CONFLICT OF INTEREST STATEMENT

The authors declare no conflict of interest.

## ETHICS STATEMENT

This trial was approved by the regional ethics committee (ID number 589‐18 and 2023‐00535‐02). Written informed consent was obtained from all subjects before the study.

## Data Availability

The data that support the findings of this study are available from the corresponding author upon reasonable request.
